# Sexual Effect of Platelet-to-Lymphocyte Ratio in Predicting Cardiovascular Mortality of Peritoneal Dialysis Patients

**DOI:** 10.1155/2022/8760615

**Published:** 2022-01-04

**Authors:** Hui Sheng, Yagui Qiu, Xi Xia, Chunyan Yi, Jianxiong Lin, Xiao Yang, Fengxian Huang

**Affiliations:** ^1^Department of Nephrology, The First Affiliated Hospital of Sun Yat-sen University, Guangzhou, 58th, Zhongshan Road II, Guangzhou 510080, China; ^2^Key Laboratory of Nephrology, National Health Commission of China and Guangdong Province, Guangzhou 510080, China

## Abstract

**Background:**

The study is aimed at exploring the relationship of platelet-to-lymphocyte (PLR), all-cause, and cardiovascular disease (CVD) mortality in peritoneal dialysis (PD) patients based on gender.

**Methods:**

A total of 1438 PD patients from January 1,2007 to December 31, 2014 in PD center at The First Affiliated Hospital, Sun Yat-sen University, were included. Patients were followed up until December 31, 2019. The endpoint was all-cause mortality and CVD mortality. Cox proportional hazards regression models were used to evaluate the association of PLR with all-cause and CVD mortality to calculate hazard ratios (HR) and 95% confidence intervals (CI).

**Results:**

After a median of 48.9 (interquartile range [IQR]: 23.4-79.3) months of follow-up, 406 (28.2%) patients died based on all-cause death, among which 200 (49.3%) patients died from CVD. In the multivariate Cox regression model, we found that PLR was independently related to an increased risk of CVD mortality only in female PD patients, with HR of 1.003 (95% CI: 1.001-1.006). Interaction test showed that the correlation between PLR level for all-cause and CVD mortality varied with gender (*p* = 0.042 and *p* = 0.012, respectively).

**Conclusion:**

Higher PLR was associated with a higher risk of CVD mortality in female PD patients.

## 1. Introduction

Chronic kidney disease (CKD) is a global problem. One in ten adults worldwide has CKD [1]. Peritoneal dialysis (PD) is a recognized method of treatment for patients who suffer from end stage renal diseases (ESRD). Inflammation is an important source of risk for the progress of CKD. It is caused by multiple factors of the toxic uremic milieu and the dialysis procedure itself [2, 3]. Microinflammation is a key component of inflammation and is closely related to lack of nutrition and atherosclerosis [4]. Some studies suggest that limiting inflammation can have important effects on halting CKD progression and reducing CVD events [4–7].

PLR is a test that reflects variations in platelet and lymphocyte levels. In many current clinical studies, PLR is considered an indicator of the systemic inflammatory response when the patient has no significant infection [8]. Higher PLR can predict poor prognosis of colorectal cancer and clinical outcomes of non-small-cell lung cancer [8–13]. Moreover, Chen et al. have established a connection between PLR and CVD disease in continuous ambulatory peritoneal dialysis patients [13]. Recently, Liu et al. reported that high PLR could predict all-cause death in PD patients [14]. Many researchers had confirmed that there were individual differences in PLR. Women had higher levels of PLR than men [15]. Regrettably, there were few studies reflecting the correlations between PLR and sex difference and the prognostic significance in patients on PD. The purpose of this scientific research was to explore the correlations between PLR and all-cause death and CVD death in patients on PD.

## 2. Materials and Methods

### 2.1. Participants

This was a retrospective cohort study. All enrolled patients came from PD center of the First Affiliated Hospital, Sun Yat-sen University, and had undergone catheterization for PD in the same center from 1 January 2007 to 31 December 2014. Exclusion criteria were the following: (1) age ≤ 18 years old; (2) patients with malignancy history or kidney transplant history; (3) the duration of PD treatment < 3 months; (4) patients had catheter insertion in other PD centers; (5) patients transferred from chronic hemodialysis (HD) > 3 months; (6) patients who had acute infection ≤ 4 weeks and had hematological disease or autoimmune disease; (7) missing platelet and lymphocyte data at baseline; and (8) patients whose biochemical parameters were obtained >3 months after PD initiation. The study was in accordance with the Declaration of Helsinki and approved by the Human Ethics Committees of Sun Yat-sen University. When participants started to receive PD treatment, they signed written informed consent.

We followed up all participants until death, transferring to HD treatment, kidney transplantation or transferring to other centers, losing connection, or the deadline of follow-up on December 31, 2019. Patients were asked to visit our PD center quarterly [16–19] for health assessment and concomitant telephone usage. Trained nurses in PD center also interviewed patients by telephone every month to assess their general health and comprehensive medical assessment. These quarterly visits and monthly calls were made for clinical purposes, not specifically for the study.

The outcomes of this research were all-cause mortality and CVD mortality. The CVD mortality referred to death from coronary events, arrhythmias, sudden cardiac death, congestive heart failure, arteriosclerosis, or cerebrovascular events. The history of CVD was defined as congestive heart failure, ischemic heart disease, cerebrovascular disease, and arteriosclerosis. Diabetes was defined according to diagnostic criteria from the American Diabetes Association. [20]

### 2.2. Clinical Data

We obtained baseline demographic, biochemical, and clinical data including age, gender, blood pressure, body mass index (BMI), history of diabetes and CVD, hemoglobin (Hb), serum albumin (Alb), plasma creatinine, serum uric acid (UA), total cholesterol, platelet, lymphocyte, estimated glomerular filtration rate (eGFR), platelet inhibitors, and *β*-blockers that were obtained during the first 1-3 month of PD. We measured all biochemical parameters in the center laboratory of the First Affiliated Hospital of Sun Yat-sen University. Among them, we measured complete blood cell count by using Sysmex XE2100 and XE5000 (Sysmex company in Kobe, Japan). We collected medication usage data according to the patients' files. Platelet inhibitor includes aspirin and clopidogre. We calculated body mass index (BMI) as follows: BMI = weight (kg)/height^2^ (m^2^). We adopted the modified simplified Modification of Diet in Renal Disease (MDRD) formula to calculate eGFR and standardize the results by using body surface area. We used a formula of eGFR = 186 × [serum creatinine]^−1.154^ × [age]^−0.203^ × [0.742 if female], and serum creatine was expressed as *μ*mol/L [21].

### 2.3. Statistical Analysis

Normal distribution of continuous variables was presented as means and standard deviations, and independent-sample *t*-test was used for the comparison between groups; skewed distributions of continuous variables were presented as medians and interquartile ranges (IQR), and the difference between groups was compared by Mann–Whitney *U* test; we presented categorical variables as number and percentages. Variables between two groups of categorical variables were compared by a *χ*^2^. Baseline PLR was evaluated as a continuous variable. We divided all patients into two groups by the median of PLR: group 1 (≤156.43) and group 2(>156.43).

We used Kaplan-Meier survival analysis to generate survival curves and examined the differences between the survival curves by using the log-rank test. We used univariate and multivariate Cox proportional hazards regression models to analyze the associations between PLR and outcomes by calculating hazard ratios (HR) and 95% confidence intervals (CI). The multivariate Cox proportional hazards regression model included variables which were identified significant association with all-cause and CVD mortality in univariate analysis (*p* < 0.1) or conventional confounding factors. The adjusted model included demographic variables (baseline age, sex, history of CVD, diabetic status) and laboratory examination (hemoglobin, serum albumin, eGFR) and clinical data (platelet inhibitor, *β*-blockers). Platelet inhibitor includes aspirin, clopidogrel, and sulodexid. We evaluated the interactions between PLR and gender and outcomes by using the multivariate Cox proportional hazards regression models. Statistical analysis was performed with SPSS software (SPSS version 25.0, SPSS Inc.). *p* < 0.05 was considered to be statistically significant.

## 3. Results

### 3.1. Baseline Clinical Data

**Figure 1:**
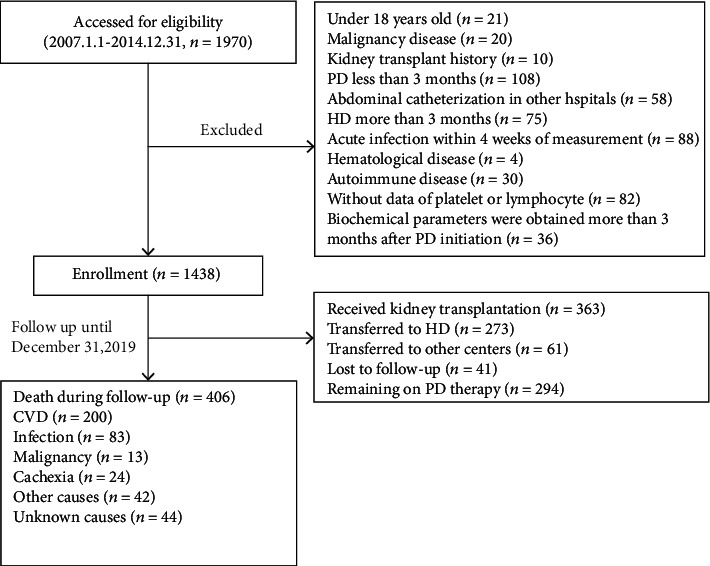
Study flow chart. Abbreviation: CVD: cardiovascular disease; PD: peritoneal dialysis; HD: hemadialysis.

The exclusionary cascade for derivation of the cohort was shown in Figure 1. In total, 1438 PD patients were included in this study. Female accounted for 39.3%. The mean age was 47.4 ± 15.3 years. The median follow-up time was 48.9 months (IQR: 23.4-79.3). The primary etiology of ESRD was glomerulonephritis (60.9%), and the second cause of ESRD was diabetic nephropathy (21.5%).

The baseline characteristics of patients according to groups of PLR level were summarized in Table 1. Compared with group 1, participants in group 2 had higher levels of PLR, as well as serum albumin, total cholesterol, platelet, and eGFR but a lower serum creatinine and lymphocyte. Participants with a PLR level > 156.43 were older, were more often women, and had a higher history of diabetes, platelet inhibitor. The median of PLR level at baseline for all patients was 156.4 (IQR: 118.1, 206.4) as the median of platelet was 232.0 × 109/L (IQR : 183.0, 288.0) × 109/L, and the median of lymphocyte was 1.5 × 109/L (IQR : 1.2, 1.8) × 109/L.

### 3.2. Factors Associated with PLR

Multivariate linear regression analysis revealed that age (*β* = 0.446, *p* = 0.010), female gender (*β* = 10.073, *p* = 0.029), diabetes (*β* = 16.280, *p* = 0.009), and serum albumin (*β* = 1.098, *p* = 0.034) were independently positively associated with PLR, after adjusting for age, gender, history of CVD and diabetes, hemoglobin, serum albumin, eGFR, platelet inhibitor, and *β*-blockers (Table 2).

### 3.3. Associations between PLR and Clinical Outcomes

The median overall survival was 48.9 (IQR: 23.4-79.3) months. By the end of this study period, 273 (19.0%) cases were transferred to HD, 363 (25.2%) cases received kidney transplantation, 61 (4.2%) cases were referred to other centers, 41 (2.9%) cases had lost follow-up, and 406 (28.2%) cases had died. Among the 406 deaths, 200 (49.3%) died from CVD events, 83 (20.4%) from infection, 13 (3.2%) from malignancy, 24 (5.9%) from cachexia, 42(10.3%) from other causes, and 44 (10.8%) from unknown causes (Figure 1).

In our study, univariate and multivariate Cox proportional hazards regression models used to analyze prognostic factors were listed in Table 3. Multivariate Cox regression analysis showed that PLR was not independently linked to all-cause death (HR: 1.000, 95% CI: 0.999-1.001). PLR was not independently linked to CVD death (HR: 1.001, 95% CI: 0.999-1.002) and ether. It showed that PLR was not independently associated with an increased risk of all-cause or CVD death.

### 3.4. PLR in Mortality Varied with Gender

Interaction tests showed that the correlations between PLR and all-cause mortality and cardiovascular mortality varied by gender (*p* = 0.042 and *p* = 0.012, respectively, Table 4).

**Figure 2:**
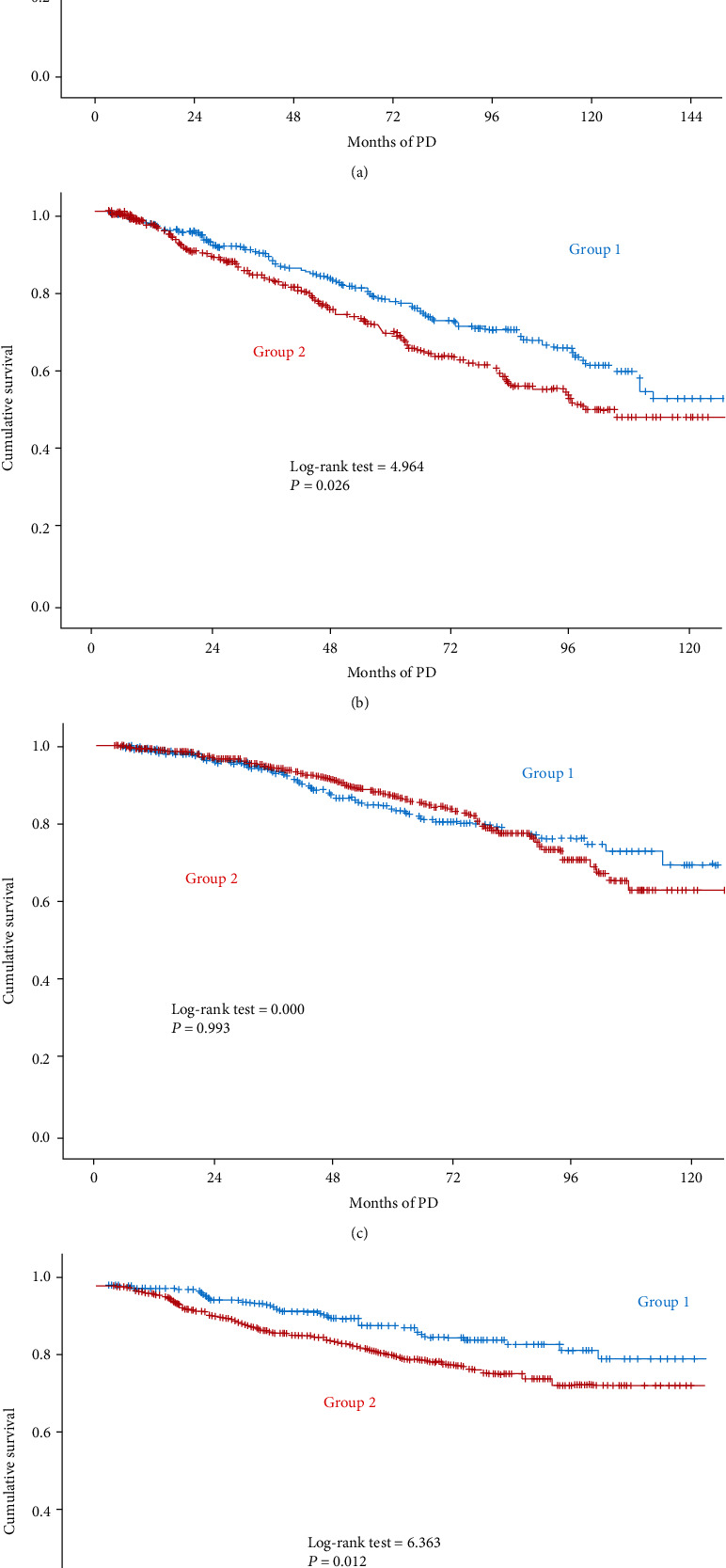
PLR and mortality stratified by sex in peritoneal dialysis (PD) patients. Cumulative risk for all-cause mortality in male patients (a) and female patients (b). Cumulative risk for cardiovascular mortality in male patients (c) and female patients (d). PLR for groups: group 1 (≤156.43) and group 2 (>156.43).

Subgroup analysis was performed to further assess the correlation of different PLR levels and death risk in gender subgroups. After applying multivariate Cox models (adjusted for all covariates), higher PLR was associated with increased risk of CVD mortality (HR: 1.003, 95% CI: 1.001-1.006; *p* = 0.008) only in the female subgroup and not among male patients (Table 4). Kaplan-Meier survival showed that higher PLR was associated with a significantly increased risk for CVD death only in female cases, but not in male cases (Figure 2).

## 4. Discussion

In this study, CVD mortality in female PD patients with higher PLR level was significantly higher than that in female PD patients with lower PLR level.

PLR may be more valuable than counting platelets or lymphocytes alone due to its reflection of both inflammation and thrombosis [22]. Some studies have reported the association of PLT and mortality. Chen et al. showed high PLR could predict the risk of CVD events rather than CVD mortality in continuous ambulatory peritoneal dialysis patients [13]. Liu et al. reported that PLR was an independent predictor of all-cause mortality in PD patients [14]. The conclusion was different from our study. It might be attributed to sample size, timing of follow-up, and the difference of multivariate Cox covariates. However, the association of PLR and gender and CVD mortality was not studied in these studies. In our study, we proved that an increased in PLR may increase the risk of CVD death in female patients on PD.

The potential mechanism for the relation between PLR and CVD death may be proposed. PLR has been considered as an indicator of systemic inflammation [9–12]. As we all know, systemic inflammation was closely related to cardiovascular mortality [4–7]. Some studies have shown that high PLR is associated with increased mortality in various disease states [23–25]. A multitude of proinflammatory cytokines is released by the activation of platelets and mediates the interaction with leukocytes, which lead to the exacerbation of inflammation [26, 27]. Inflammation becomes ubiquitous among PD patients [4, 14]. Both acute peritonitis and microinflammation are important constituents of systemic inflammation responses [28–30]. In PD patients, microinflammation may be associated with the accumulation of uremic wastes, PD catheterization, bioincompatible dialysate, and periodontal problems [28, 31]. Microinflammation is also an important part of systemic inflammatory response. As we known, the association among inflammation, malnutrition, and atherosclerosis has been described as malnutrition, inflammation, and atherosclerosis (MIA) syndrome. These three factors influence one another and eventually leading to increased CVD death [29–32].

Furthermore, interaction analysis suggested that gender was the most important effect modifier in this study. In subgroup analysis, PLR was independently associated with higher mortality of cardiovascular diseases only in female patients. Some scholars have reported the sex difference in PLR. Lee et al. found that PLR was higher in women than that in men according to their study of more than 10,000 patients from a single racial group [15]. However, another research from Central China reported that there was no difference between male and female in PLR [33]. A study showed that high baseline PLR could predict poor renal survival in patients with IgAN, especially in female cases [22]. In our study, we found that the sex difference in PLR may influence the effects of PLR on mortality in PD patients.

Several potential mechanisms for the association between PLR and sex difference may be proposed. Firstly, platelets and lymphocytes arise from the same hematopoietic stem cells; so, PLR remains constant to keep the balance in vivo [34, 35]. An elevated level of PLR may represent relatively high platelets and low lymphocytes. Low numbers of lymphocytes may reveal immune dysfunction or a weakened defense [36, 37]. Gender difference may be related to higher platelet count in women. Estradiol synthesized in megakaryocytes triggers proplatelet formation (PPF) and then regulated platelet production [38]. While women have more estrogen than men, PLR may be higher than men. Secondly, low levels of serum iron in female may be induced by regular menstruation, which increases platelet production [39, 40]. Taking these factors together, it is not surprising that PLR was an independent risk factor for CVD death in female PD patients.

The advantages of this research include its complete data on clinical outcomes. However, there were several limitations to our research. First, the lack of follow-up data is a major limitation. There is a need for longitudinal studies to explore whether the relationship between PLR and mortality changes over time. Second, some factors that may be related to PLR and mortality are not discussed in this study because peritoneal equilibration test data of some patients are missing. Third, this is a single-center study of Chinese patients with peritoneal dialysis, implying limited generalizability.

## 5. Conclusions

PLR level was an independent risk factor for CVD mortality only in female PD patients. The current study reveals that we should pay close attention to PLR in PD patients, especially in female patients. Regular evaluating and monitoring are essential.

## Figures and Tables

**Table 1:** Baseline characteristics of the study cohort.

Variable	Total (*n* = 1438)	Group 1 (*n* = 719)	Group 2 (*n* = 719)	*p* value
Age (years)	47.4 ± 15.3	46.4 ± 15.4	48.4 ± 15.2	0.011
Gender (female, *n*, %)	565 (39.3)	260 (36.2)	305 (42.4)	0.015
BMI (kg/m^2^)	21.6 ± 3.1	21.6 ± 3.0	21.6 ± 3.2	0.944
SBP (mmHg)	134.2 ± 20.0	133.5 ± 20.2	134.9 ± 19.7	0.203
DBP (mmHg)	84.6 ± 23.6	84.5 ± 15.3	84.8 ± 29.3	0.830
History of CVD (*n*, %)	246 (17.1)	115 (16.0)	131 (18.2)	0.263
Diabetes (*n*, %)	297 (20.7)	131 (18.2)	166 (23.1)	0.023
HGB (g/L)	101.4 ± 20.7	100.6 ± 20.8	102.3 ± 20.6	0.117
ALB (g/L)	36.9 ± 4.9	36.5 ± 4.9	37.2 ± 5.0	0.018
Plasma creatinine (*μ*mol/L)	739.3 ± 293.3	758.8 ± 314.7	719.9 ± 269.0	0.012
UA (*μ*mol/L)	422.5 ± 94.2	425.0 ± 91.7	419.9 ± 96.7	0.315
Total cholesterol (mmol/L)	5.1 ± 1.8	4.9 ± 1.2	5.2 ± 2.2	0.003
Platelet (×10^9^/L)	232.0 (183.0, 288.0)	194.0(154.0,243.0)	271.0(222.0,319.0)	<0.001
Lymphocyte (×10^9^/L)	1.5 (1.2, 1.8)	1.7 (1.4, 2.1)	1.2 (1.0, 1.5)	<0.001
PLR	156.4 (118.1, 206.4)	118.1 (96.3, 136.1)	206.4 (178.9, 254.6)	<0.001
eGFR (mL/min/1.73 m^2^)	6.8(5.3, 8.8)	6.7 (5.1, 8.8)	7.0(5.5, 8.8)	0.060
Platelet inhibitor (*n*, %)	85 (5.9)	32 (4.5)	53 (7.4)	0.019
*β*-Blockers (*n*, %)	676 (47.0)	325 (45.2)	351 (48.8)	0.170

Abbreviations: BMI: body mass index; SBP: systolic blood pressure; DBP: diastolic blood pressure; CVD: cardiovascular disease; HGB: hemoglobin; ALB: serum albumin; UA: uric acid; PLR: the platelet-to-lymphocyte ratio; eGFR: estimated glomerular filtration rate; PLR: for groups: group 1 (≤156.43) and group 2(>156.43).

**Table 2:** Multiple linear regression analysis on influencing factors of PLR.

Variable	Unstandardized regression coefficient		Standardized regression coefficient	*T*	*p* value
	*B*	Standard error			
Age (y)	0.446	0.173	0.082	2.574	0.010
Gender (female)	10.073	4.619	0.059	2.181	0.029
History of CVD (yes/no)	-0.758	6.457	-0.003	-0.117	0.907
Diabetes (yes/no)	16.280	6.229	0.080	2.614	0.009
HGB (g/L)	0.085	0.120	0.021	0.705	0.481
ALB (g/L)	1.098	0.518	0.065	2.121	0.034
eGFR (mL/min/1.73 m^2^)	-0.063	0.568	-0.003	-0.110	0.912
Platelet inhibitor (yes/no)	12.695	9.878	0.036	1.285	0.199
*β*-Blockers (yes/no)	4.035	4.531	0.024	0.891	0.373

*F* = 3.862, *p* < 0.001, *R*^2^ = 0.025. Analysis of factors associated with PLR by multivariable linear regression. Abbreviations: PLR: the platelet-to-lymphocyte ratio; CVD: cardiovascular disease; HGB: hemoglobin; ALB: serum albumin; eGFR: estimated glomerular filtration rate.

**Table 3:** Associations of PLR with all-cause and CVD mortality in univariate and multivariate Cox regression models^∗^.

Variable	All-cause mortality unadjusted model		Multivariate model^∗^		Cardiovascular mortality unadjusted model		Multivariate model^∗^	
	HR (95% CI)	*p* value	HR (95% CI)	*p* value	HR (95% CI)	*p* value	HR (95% CI)	*p* value
Age (y)	1.063 (1.056-1.071)	<0.001	1.056 (1.047-1.065)	<0.001	1.065 (1.054-1.076)	<0.001	1.054 (1.041-1.068)	<0.001
Gender (female, *n*, %)								
Female	0.978 (0.804-1.191)	0.826	1.001 (0.817-1.226)	0.995	0.877 (0.661-1.166)	0.367	0.863 (0.640-1.163)	0.333
Male	Ref.		Ref.		Ref.		Ref.	
History of CVD (yes/no)								
Yes	2.771 (2.242-3.425)	<0.001	1.373 (1.089-1.731)	0.007	3.341 (2.496-4.471)	<0.001	1.655 (1.200-2.282)	0.002
No	Ref.		Ref.		Ref.		Ref.	
Diabetes (yes/no)								
Yes	3.299 (2.706-4.021)	<0.001	1.650 (1.327-2.051)	<0.001	3.911 (2.959-5.171)	<0.001	1.999 (1.460-2.737)	<0.001
No	Ref.		Ref.		Ref.		Ref.	
HGB (g/L)	0.990 (0.985-0.994)	<0.001	0.990 (0.985-0.996)	<0.001	0.991 (0.984-0.997)	0.006	0.990 (0.982-0.998)	0.015
ALB (g/L)	0.913 (0.895-0.932)	<0.001	0.972 (0.950-0.996)	0.020	0.927 (0.901-0.955)	<0.001	0.994 (0.960-1.029)	0.730
eGFR (mL/min/1.73 m^2^)	1.022 (1.013-1.031)	<0.001	1.017 (1.003-1.032)	0.020	1.018 (1.003-1.034)	0.017	1.000 (0.963-1.039)	0.999
PLR	1.001 (1.000-1.003)	0.005	1.000 (0.999-1.001)	0.594	1.002 (1.001-1.004)	0.002	1.001 (0.999-1.002)	0.250
Platelet inhibitor (yes/no)								
Yes	1.811 (1.301-2.520)	<0.001	0.893 (0.628-1.270)	0.529	2.077 (1.333-3.236)	0.001	0.936 (0.583-1.503)	0.784
No	Ref.		Ref.		Ref.		Ref.	
*β*-Blockers								
(yes/no)								
Yes	0.683 (0.560-0.833)	<0.001	0.850 (0.692-1.044)	0.121	0.714 (0.538-0.946)	0.019	0.862 (0.644-1.153)	0.316
No	Ref.		Ref.		Ref.		Ref.	

^∗^All the models were adjusted for age, gender, history of CVD, diabetic status, hemoglobin, serum albumin, estimated glomerular filtration rate, platelet inhibitor, and *β*-blockers. Abbreviations: PLR: the platelet-to-lymphocyte ratio; CVD: cardiovascular disease; HGB: hemoglobin; ALB: serum albumin; PLR: the platelet-to-lymphocyte ratio; eGFR: estimated glomerular filtration rate.

**Table 4:** Interaction tests of PLR and gender and mortality, and the sexual difference in the associations between PLR and mortality^∗^.

Variables	HR	All-cause mortality		HR	Cardiovascular mortality	
		95% CI	*p* value		95% CI	*p* value
Interaction analysis	For mortality in entire cohort			*p* for interaction = 0.012		
	*p* for interaction = 0.042					
PLR × gender						
Female (*n*)	No.of all − cause deaths = 177			No.of cardiovascular deaths = 79		
PLR	1.002	1.000-1.003	0.098	1.003	1.001-1.006	0.008
Male (*n*)	No.of all − cause deaths = 229			No.of cardiovascular deaths = 121		
PLR	1.000	0.998-1.001	0.562	1.000	0.998-1.001	0.623

^∗^All the models were adjusted for age, history of CVD, diabetic status, hemoglobin, serum albumin, estimated glomerular filtration rate, platelet inhibitor, and *β*-blockers. Abbreviations: PLR: the platelet-to-lymphocyte ratio.

## Data Availability

The clinical data used to support the findings of the study are available from the corresponding author or the PD center at the First Affiliated Hospital, Sun Yat-sen University upon request.
